# Pyridoxine Deficiency Exacerbates Neuronal Damage after Ischemia by Increasing Oxidative Stress and Reduces Proliferating Cells and Neuroblasts in the Gerbil Hippocampus

**DOI:** 10.3390/ijms21155551

**Published:** 2020-08-04

**Authors:** Hyo Young Jung, Woosuk Kim, Kyu Ri Hahn, Min Soo Kang, Tae Hyeong Kim, Hyun Jung Kwon, Sung Min Nam, Jin Young Chung, Jung Hoon Choi, Yeo Sung Yoon, Dae Won Kim, Dae Young Yoo, In Koo Hwang

**Affiliations:** 1 Department of Anatomy and Cell Biology, College of Veterinary Medicine, and Research Institute for Veterinary Science, Seoul National University, Seoul 08826, Korea; hyoyoung@snu.ac.kr (H.Y.J.); hkinging@snu.ac.kr (K.R.H.); ysyoon@snu.ac.kr (Y.S.Y.); 2 Department of Biomedical Sciences, and Research Institute for Bioscience and Biotechnology, Hallym University, Chuncheon 24252, Korea; tank3430@hallym.ac.kr; 3 Department of Anatomy, College of Veterinary Medicine and Institute of Veterinary Science, Kangwon National University, Chuncheon 24341, Korea; imkangms@kangwon.ac.kr (M.S.K.); xogudsla9402@kangwon.ac.kr (T.H.K.); jhchoi@kangwon.ac.kr (J.H.C.); 4 Department of Biochemistry and Molecular Biology, Research Institute of Oral Sciences, College of Dentistry, Gangneung-Wonju National University, Gangneung 25457, Korea; donuts25@gwnu.ac.kr (H.J.K.); kimdw@gwnu.ac.kr (D.W.K.); 5 Department of Anatomy, College of Veterinary Medicine, Konkuk University, Seoul 05030, Korea; lovingvet@gmail.com; 6 Department of Veterinary Internal Medicine and Geriatrics, College of Veterinary Medicine, Kangwon National University, Chuncheon 24341, Korea; jychung77@gmail.com; 7 Department of Anatomy, College of Medicine, Soonchunhyang University, Cheonan 31151, Korea

**Keywords:** pyridoxine deficiency, ischemia, gerbil, homocysteine, cell death, glia, neurogenesis

## Abstract

We investigated the effects of pyridoxine deficiency on ischemic neuronal death in the hippocampus of gerbil (*n* = 5 per group). Serum pyridoxal 5′-phosphate levels were significantly decreased in Pyridoxine-deficient diet (PDD)-fed gerbils, while homocysteine levels were significantly increased in sham- and ischemia-operated gerbils. PDD-fed gerbil showed a reduction in neuronal nuclei (NeuN)-immunoreactive neurons in the medial part of the hippocampal CA1 region three days after. Reactive astrocytosis and microgliosis were found in PDD-fed gerbils, and transient ischemia caused the aggregation of activated microglia in the stratum pyramidale three days after ischemia. Lipid peroxidation was prominently increased in the hippocampus and was significantly higher in PDD-fed gerbils than in Control diet (CD)-fed gerbils after ischemia. In contrast, pyridoxine deficiency decreased the proliferating cells and neuroblasts in the dentate gyrus in sham- and ischemia-operated gerbils. Nuclear factor erythroid-2-related factor 2 (Nrf2) and brain-derived neurotrophic factor (BDNF) levels also significantly decreased in PDD-fed gerbils sham 24 h after ischemia. These results suggest that pyridoxine deficiency accelerates neuronal death by increasing serum homocysteine levels and lipid peroxidation, and by decreasing Nrf2 levels in the hippocampus. Additionally, it reduces the regenerated potentials in hippocampus by decreasing BDNF levels. Collectively, pyridoxine is an essential element in modulating cell death and hippocampal neurogenesis after ischemia.

## 1. Introduction

Vitamin B_6_ vitamers consist of pyridine derivatives such as naïve and phosphorylated forms of pyridoxine, pyridoxal, and pyridoxamine. Intakes of B_6_ vitamers are absorbed in the intestine and transformed into its active form, pyridoxal 5′-phosphate (PLP), in the liver. However, among B_6_ vitamers, naïve forms can cross the blood–brain barrier in the brain [1]. PLP acts as a coenzyme or cofactor in more than 100 reactions associated with energy metabolism and neurotransmitter synthesis. In addition, vitamin B_6_ has antioxidant properties that quench reactive oxygen [2] and reduce the formation of advanced glycation end-products [3,4].

Brain ischemia is one of the major life-threatening diseases worldwide, and it debases the quality of life in survivors. Mongolian gerbils (*Meriones unguiculatus*) are used in animal models for brain ischemia because they have incomplete posterior communicating arteries that cause brain ischemia only by occlusion of the common carotid artery for 5 min in the neck region [5]. However, more sophisticated models with complete interruption of cerebral blood flow have been implemented in rats [6,7,8] and mice [9], which are more widely used in research. Several studies demonstrate that PLP has neuroprotective effects against various neurological diseases including ischemia [10,11], vascular dementia [12], Parkinson’s disease [13], and cortical damage [14]. In addition, pyridoxine treatment increases proliferating cells and neuroblasts in the dentate gyrus [15]. There is conflicting evidence of dietary vitamin B_6_ on cardiovascular diseases in human studies [16,17,18,19]. Plasma vitamin B_6_ levels are inversely related to risk and incidence of cardiovascular diseases in the United States, Japan, and Korea [16,17,18], while a Finnish study demonstrates that there is no relationship between vitamin B_6_ intake and cardiovascular diseases [19].

In contrast, feeding with pyridoxine deficient diets causes cognitive impairment in normal mice [20] and in animal models for Alzheimer’s disease [21]. Additionally, pyridoxine deficiency increases homocysteine levels because pyridoxine is used in homocysteine metabolism [22]. Increased homocysteine is considered as a risk factor for stroke [23], and it facilitates the production of reactive oxygen species by auto-oxidation [24]. However, there are no studies on the effects of pyridoxine deficiency on neuronal cell death after ischemia in the hippocampus. In this study, we examined the effects of pyridoxine deficiency on ischemia-induced cell death based on the oxidative stress in the hippocampus after 5 min of forebrain ischemia.

## 2. Results

### 2.1. Pyridoxine Deficiency Decreases PLP and Increases Homocysteine Levels in Serum

**Figure 1:**
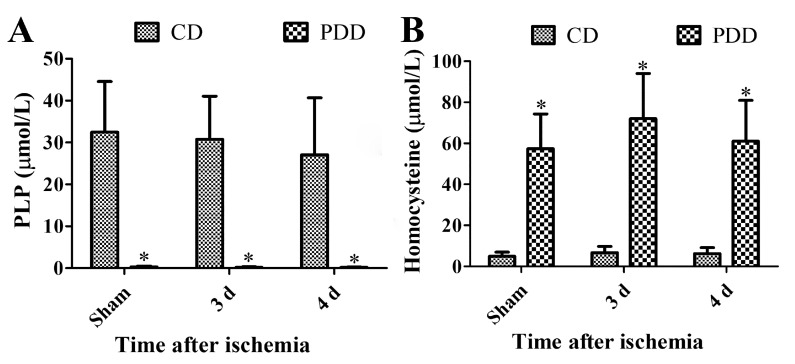
Pyridoxine deficiency decreases serum pyridoxal 5′-phosphate (PLP) levels (**A**) and increases serum homocysteine levels (**B**) in the control diet (CD)- and pyridoxine-deficient diet (PDD)-fed gerbils of sham- and ischemia-operated groups. Data were analyzed with a two-way ANOVA test followed by Bonferroni post-hoc tests (*n* = 7 per group; * *p* < 0.05, significant difference between CD- and PDD-fed group). All data are expressed as mean with standard deviation.

In the Control diet (CD)-fed sham group, PLP and homocysteine levels were 32.5 ± 12.1 μmol/L and 4.91 ± 2.05 μmol/L in the serum, respectively. In the Pyridoxine-deficient diet (PDD)-fed sham group, PLP levels were dramatically decreased to 0.298 ± 0.205 μmol/L, while homocysteine levels were significantly increased to 57.4 ± 16.9 μmol/L. Transient forebrain ischemia decreased PLP levels in CD- and PDD-fed groups, although the statistical significance was not detected between CD- and PDD-fed groups. PLP levels were significantly lower in the PDD-fed group compared to the CD-fed group three and four days after ischemia. In contrast, homocysteine levels were maintained with significant increases in PDD-the fed group compared to that in the CD-fed group three and four days after ischemia, although homocysteine levels were slightly higher after ischemia compared to that in the sham group. The two-way analysis of variance (ANOVA) test showed that there were no interactions between ischemia and PDD diets in PLP and homocysteine levels in the serum (Figure 1A,B).

### 2.2. Pyridoxine Deficiency Causes Early Neuronal Death after Ischemia

**Figure 2:**
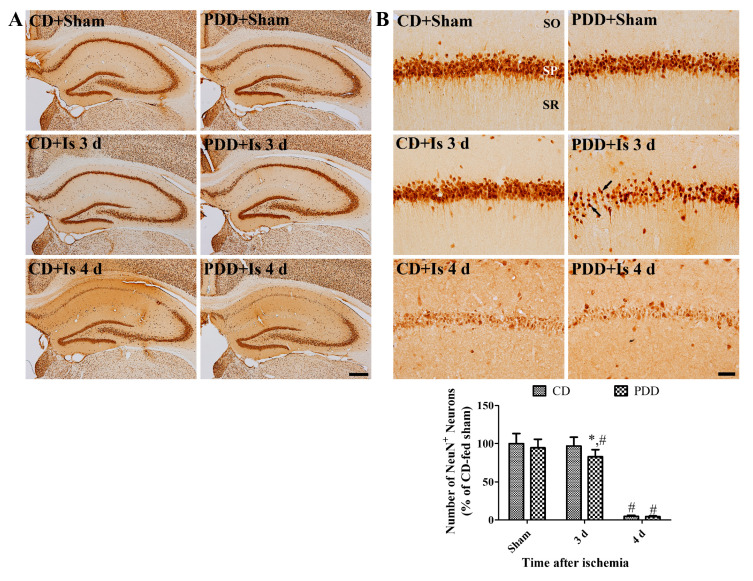
Immunohistochemical staining for neuronal nuclei (NeuN) in the whole hippocampus (**A**) and its magnified CA1 region (**B**) in the CD- and PDD-fed gerbils of sham- and ischemia-operated groups. Note that fewer NeuN-positive cells (arrows) are found in the medial side of the stratum pyramidale (SP) in the PDD-fed gerbils three days after ischemia. SO, stratum oriens; SR, stratum radiatum. Scale bar = 400 μm (**A**), 50 μm (**B**). The number of NeuN-immunoreactive nuclei in the CA1 region compared to the CD-fed sham group per section, in all the groups, is shown (*n* = 7 per group; * *p* < 0.05, significant difference between CD- and PDD-fed group; ^#^ *p* < 0.05, significant difference between sham- and ischemia-operated groups). All data are expressed as mean with standard deviation.

In the CD-fed sham group, neuronal nuclei (NeuN)-positive neurons were abundantly observed in all hippocampal regions, including the CA1 region, and the same observation was also obtained in the PDD-fed sham group. In the PDD-fed sham group, the number of NeuN-positive neurons was slightly decreased to 94.8% of the CD-fed sham group. In the CD-fed ischemic group, numerous NeuN-positive neurons were found in the hippocampus three days after ischemia, while NeuN-positive neurons were decreased in the medial side of the CA1 region and not in the lateral region three days after ischemia. The number of NeuN positive neurons was significantly decreased in the PDD-fed group compared to that in the CD-fed sham group to 83.2% of the CD-fed sham group. Four days after ischemia, NeuN-positive neurons were prominently decreased in the hippocampal CA1 region of the CD- and PDD-fed ischemia group to 4.9% and 4.4% of the CD-fed sham group. However, there were no significant differences in the number of NeuN-positive neurons between groups. The two-way ANOVA test demonstrated that there were no interactions between ischemia and PDD diets in neuronal numbers in the hippocampal CA1 region (Figure 2A,B).

### 2.3. Pyridoxine Deficiency Facilitates the Activation of Astrocytes and Microglia after Ischemia

**Figure 3:**
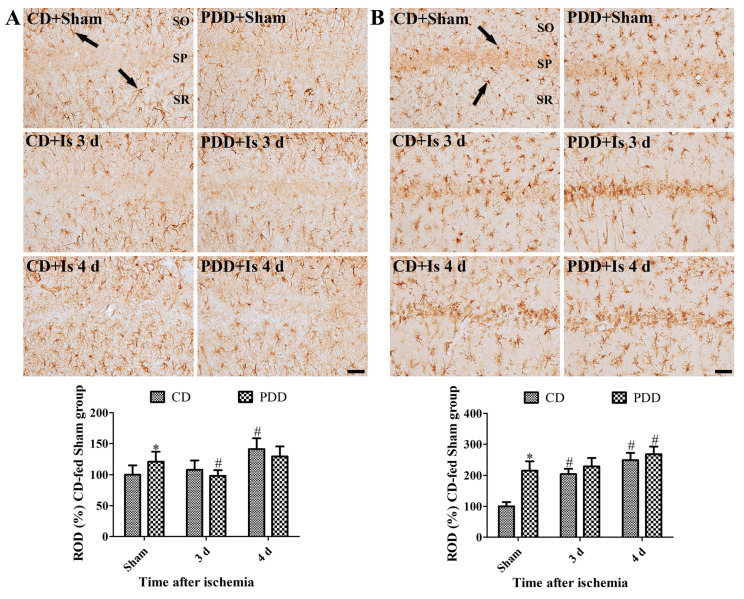
Immunohistochemical staining for GFAP (**A**) and Iba-1 (**B**) in the hippocampal CA1 region in the CD- and PDD-fed gerbils of sham- and ischemia-operated groups. Note that GFAP-immunoreactive astrocytes (arrows) and Iba-1-immunoreactive microglia (arrows) show morphological changes after PDD-diet and ischemic damage. SO, stratum oriens; SP, stratum pyramidale; SR, stratum radiatum. Scale bar = 50 μm. Relative optical density (ROD) corresponding to the percentage of GFAP and Iba-1 immunoreactivity value in the hippocampal CA1 region of the CD-fed sham group per section is shown (*n* = 7 per group; * *p* < 0.05, significant difference between CD- and PDD-fed groups; ^#^ *p* < 0.05, significant difference between sham- and ischemia-operated groups). All data are expressed as mean with standard deviation.

In the CD-fed sham group, glial fibrillary acidic protein (GFAP)-immunoreactive astrocytes and ionized calcium-binding adapter molecule 1 (Iba-1)-immunoreactive microglia had small cytoplasm with long and distinct processes. In the PDD-fed sham group, they had enlarged cytoplasm with thickened processes in the hippocampal CA1 region, and GFAP and Iba-1 immunoreactivities were significantly increased in this group, compared to that in the CD-fed sham group. Three days after ischemia/reperfusion, GFAP-immunoreactive astrocytes showed similar morphology in CD- and PDD-fed groups compared to the PDD-fed sham group, and GFAP immunoreactivity was similarly observed. In contrast, Iba-1-immunoreactive microglia showed similar morphology in CD-fed ischemic group three days after ischemia/reperfusion, but in the PDD-fed group, Iba-1-immunoreactive microglia were abundantly found in the medial side of the stratum pyramidale, where the neuronal death occurred. Additionally, Iba-1-immunoreactive microglia that were detected in the stratum pyramidale had round cytoplasm without processes, and Iba-1 immunoreactivity was significantly increased. Four days after ischemia/reperfusion, GFAP-immunoreactive astrocytes had punctuated cytoplasm with thickened processes in CD- and PDD-fed groups. Iba-1-immunoreactive microglia were abundantly observed in the stratum pyramidale of the CA1 region as well as the stratum oriens and radiatum in CD- and PDD-fed groups. There were no significant differences in the GFAP and Iba-1 immunoreactivities between CD- and PDD-fed groups after ischemia. However, the two-way ANOVA test indicated that ischemia and PDD diets significantly increased the GFAP (df = 2, F = 5.341, *p* = 0.0093) and Iba-1 immunoreactivity (df = 2, F = 19.06, *p* < 0.0001) (Figure 3A,B).

### 2.4. Pyridoxine Deficiency Decreases Ischemia-Induced Proliferating Cells and Neuroblasts

**Figure 4:**
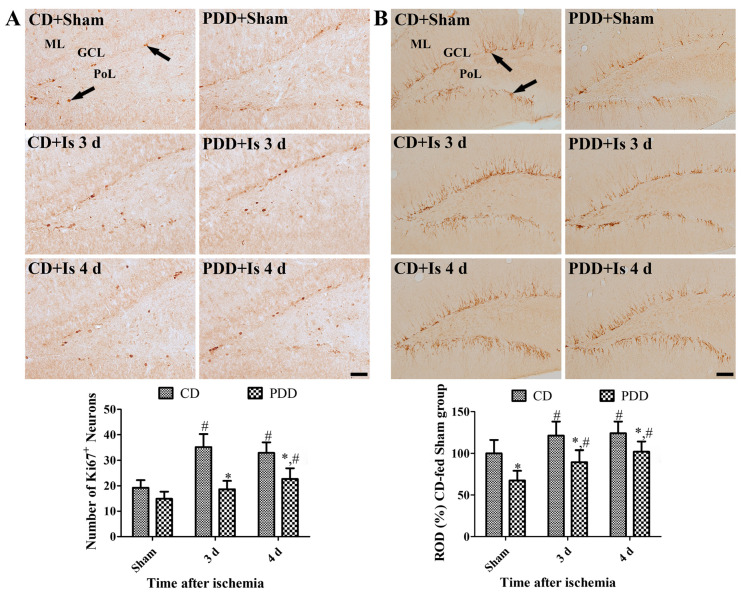
Immunohistochemical staining for Ki67 (**A**) and doublecortin (DCX) (**B**) in the dentate gyrus in the CD- and PDD-fed gerbils of sham- and ischemia-operated groups. Note that fewer proliferating Ki67-positive cells (arrows) and lower DCX immunoreactivity (arrows) are found in the PDD-fed gerbils than in the CD-fed ones. SO, stratum oriens; SP, stratum pyramidale; SR, stratum radiatum. Scale bar = 50 μm (**A**) and 100 μm (**B**). The number of Ki67-positive nuclei in the dentate gyrus and the ROD of DCX compared to the CD-fed sham group per section, in all the groups, are shown (*n* = 7 per group; * *p* < 0.05, significant difference between CD- and PDD-fed group; ^#^ *p* < 0.05, significant difference between sham- and ischemia-operated groups). All data are expressed as mean with standard deviation.

In all groups, Ki67-positive nuclei and cell bodies of doublecortin (DCX)-immunoreactive neuroblasts were mainly found in the subgranular zone of the dentate gyrus, and processes of neuroblasts branched out to the molecular layer of the dentate gyrus. However, there were significant changes in the number of proliferating Ki67-positive cells and the immunoreactivity of DCX-immunoreactive neuroblasts among groups. In the sham group, the DCX immunoreactivity was significantly decreased in the PDD-fed group compared to that in the CD-fed group, while the number of Ki67-positive cells was slightly decreased. Three and four days after ischemia/reperfusion, the number of Ki67-positive cells and the DCX immunoreactivity were significantly increased compared to the sham group, and there were fewer Ki67-positive cells and lower DCX immunoreactivity in the PDD-fed group compared to that in the CD-fed group. However, the two-way ANOVA test indicated that ischemia and PDD diets significantly changes the number of proliferating Ki67-positive cells (df = 2, F = 8.816, *p* = 0.0008), and there were no interaction effects of ischemia and PDD diets in the density of DCX-immunoreactive neuroblasts (Figure 4A,B).

### 2.5. Pyridoxine Deficiency Increases Lipid Peroxidation and Decreases Nrf2 and BDNF Expression

**Figure 5:**
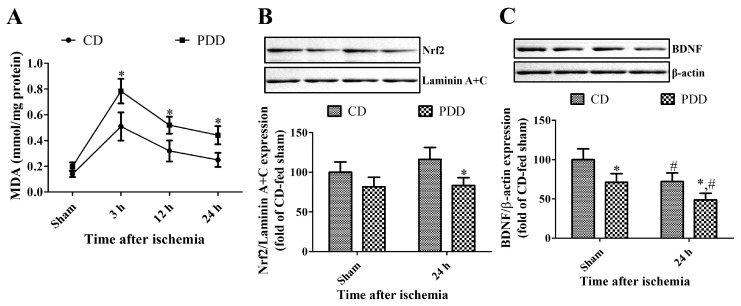
Measurement of malondialdehyde (MDA) levels (**A**) in the hippocampus and Western blot analysis of nuclear Nrf2 (**B**) and total mature brain-derived neurotrophic factor (BDNF) levels (**C**) in the CD- and PDD-fed gerbils of sham- and ischemia-operated groups (*n* = 6–7 per group; * *p* < 0.05, significant difference between CD- and PDD-fed groups; ^#^ *p* < 0.05, significant difference between sham- and ischemia-operated groups). All data are expressed as mean with standard deviation.

In the PDD-fed sham group, malondialdehyde (MDA) levels in the hippocampus were higher (43.5%) compared to that in the CD-fed sham group. MDA levels in CD- and PDD-fed groups were dramatically increased three hours after ischemia to 368.0% and 395.0% of their respective sham gerbils and thereafter, MDA levels decreased with time after ischemia in both CD- and PDD-fed groups. Twenty-four hours after ischemia, MDA levels in CD- and PDD-fed groups showed 180.4% and 222.5% of their respective sham group. MDA levels were significantly higher in the PDD-fed group compared to that in the CD-fed group after ischemia, and this was not observed in the sham group (Figure 5A).

In the PDD-fed sham group, nuclear factor erythroid-2-related factor 2 (Nrf2) levels in nuclear fraction decreased compared to that in the CD-fed sham group, although statistical significance was not detected between groups. Twenty-four hours after ischemia, nuclear Nrf2 levels were slightly, but not significantly, increased in the CD-fed group, but they were maintained in the PDD-fed group. At this time point, nuclear Nrf2 showed significantly lower levels (71.6% of the CD-fed group) in the PDD-fed group (Figure 5B).

In the sham group, brain-derived neurotrophic factor (BDNF) levels in the PDD-fed gerbils were significantly decreased to 71.1% of CD-fed gerbils. Moreover, BDNF levels were significantly decreased in the hippocampus 24 h after ischemia/reperfusion in CD- and PDD-fed gerbils, and significantly lower levels (67.5% of CD-fed gerbils) of BDNF were found in the PDD-fed gerbils 24 h after ischemia (Figure 5C).

The two-way ANOVA test indicated that ischemia and PDD diets significantly changed MDA levels (df = 3, F = 3.807, *p* = 0.0193) in the hippocampus, while there were no interactive effects of ischemia and PDD diets on Nrf2 and BDNF expression in the hippocampus.

## 3. Discussion

Vitamin B_6_ is one of the essential vitamins that maintain the health of the nervous system in mammals, which should be taken in sufficient quantity from the food because it is not synthesized in mammals [25,26]. Deficiency of vitamin B_6_ causes cognitive function impairment, hippocampal neurogenesis reduction, cardiovascular disease, and Alzheimer’s disease [20,27]. In the present study, we observed the effects of pyridoxine deficiency on cell death in the hippocampal CA1 region and on neuroblasts in the dentate gyrus after transient forebrain ischemia. We observed the changes in serum levels of PLP and homocysteine because vitamins B_6_, B_12_, and folic acid have been reported to be associated with the regulation of the methionine–homocysteine cycle [22]. PDD-fed gerbils showed a significant reduction in serum PLP levels in sham-operated animals as well as ischemic animals three and four days after ischemia. This result is consistent with our previous study that PDD-fed mice showed a significant reduction in serum and hippocampal PLP levels [20]. In contrast, pyridoxine deficiency significantly increased serum homocysteine levels in sham- and ischemia-operated animals and showed hyperhomocysteinemia, which is characterized by > 15 µmol/L homocysteine levels in the blood. Vitamin B_6_, B_12_, and folic acid are associated with homocysteine metabolism [18], and our previous study showed the presence of hyperhomocysteinemia in folic acid deficient gerbils, and this condition increased the DNA damage in the hippocampus after ischemia [28]. Moreover, many studies demonstrated that hyperhomocysteinemia caused neuronal damage, cognitive impairments, and psychiatric diseases [29,30,31,32,33].

To confirm whether pyridoxine deficiency facilitates neuronal death in the hippocampal CA1 region, we conducted immunohistochemical staining of NeuN, a marker for mature neurons, in the hippocampus three and four days after ischemia because neuronal death was detected in the CA1 region four days after ischemia. In the sham group, NeuN-positive neurons slightly decreased in the CA1 region, although there is no statistical significance between the CD- and PDD-fed gerbils. A study showed that hyperhomocysteinemia significantly increased neuronal death in the rat hippocampus [34]. In the present study, we observed that pyridoxine deficiency significantly reduced the number of NeuN-positive neurons in the medial side of the CA1 region, and this suggests that pyridoxine deficiency facilitates neuronal death in the CA1 region after ischemia. However, we did not observe any significant changes in the number of NeuN-positive nuclei four days after ischemia in CD- and PDD-fed gerbils because most of the neurons showed degeneration in the CA1 region. Pyridoxine deficiency also showed activation of astrocytes and microglia in the hippocampus of sham-operated gerbils, and microglial aggregation was found in the medial side of the stratum pyramidale three days after ischemia/reperfusion because of neuronal death in this region. These results suggest that pyridoxine deficiency facilitates glial activation in the hippocampal CA1 region of sham- and ischemia-operated gerbils three days after ischemia. Our study provides new insight into the relationship between pyridoxine deficiency and glial activation in the hippocampus. We could not elucidate the mechanisms of glial activation in the hippocampus of PDD-fed gerbils. One hypothesis is hyperhomocysteinemia induced by pyridoxine deficiency because treatment with homocysteine significantly increased reactive gliosis in astrocytes and microglia in rats [35], and excess homocysteine causes toxicity in the brain [36,37].

We examined the possible mechanisms of pyridoxine deficiency in the hippocampal neuronal death after ischemia based on oxidative stress due to the presence of antioxidant potentials in vitamin B_6_, which can quench hydroxyl radicals similar with vitamin C. In contrast, transient forebrain ischemia increases oxidative stress, including DNA damage and lipid peroxidation in the hippocampus [24,38,39,40,41]. In the present study, lipid peroxidation measured by MDA levels was significantly increased 3 h after ischemia in the CD-fed gerbils and thereafter decreased with time after ischemia. This result is consistent with previous studies that MDA products increased as an accumulation of aldehyde in various neurological disorders such as Alzheimer’s disease, ischemia, and Parkinson’s disease [24,40,41,42,43,44,45]. Additionally, MDA levels in this study were significantly higher in PDD-fed gerbils than in CD-fed ones 3, 12, and 24 h after ischemia, and not in the sham-operated group. This result suggests that PDD increases reactive aldehyde in the hippocampus, which aggravates neuronal death in the hippocampal CA1 region after transient forebrain ischemia. Changes in Nrf2 expression were also observed in the hippocampus because it is believed to be a key transcription factor in decreasing reactive oxygen species [46]. There have been reports that Nrf2 decreased ischemic damage by reducing oxidative stress [47], while Nrf2 deficient mice showed vulnerability to oxidative stress [48]. In the present study, lower Nrf2 expression levels were found in the PDD-fed gerbils than in the CD-fed gerbils 24 h after ischemia, and this result suggests that the reduction of Nrf2 may be associated with early neuronal damage after transient forebrain ischemia.

In this study, we also observed the effects of pyridoxine deficiency on regenerative potentials based on proliferating cells and neuroblasts in the dentate gyrus because we observed the reduction of neuroblasts in the mice hippocampus [20]. In gerbil brain, the proliferating cells and neuroblasts were reduced in the dentate gyrus of sham- and ischemia-operated animals, and this result suggests that pyridoxine deficiency reduces the regeneration potentials in ischemic brain. Additionally, we observed BDNF levels in the hippocampus because it modulates the hippocampal neurogenesis [49,50]. Pyridoxine deficiency and/or brain ischemia decreased BDNF levels in the hippocampus, and BDNF levels were lowest in PDD-fed ischemic gerbils. This result supports the immunohistochemical finding for proliferating cells and neuroblasts in the dentate gyrus. Interestingly, Nrf2 is one of the essential elements to regulate the hippocampal neurogenesis [51] and homocysteine reduced cell differentiation in chicken embryonic brain [52]. However, neurogenesis in the dentate gyrus is unlikely to be related to regeneration; rather, migrating neuroblasts from SVZ may contribute to regeneration [53,54]. Moreover, the initial increase in neurogenesis in the dentate gyrus may be accompanied by its significant decrease at a later stage after ischemia [7] due to the limited pool of stem cells in this region [55].

In conclusion, pyridoxine deficiency facilitates neuronal death in the hippocampal CA1 region after transient forebrain ischemia by increasing homocysteine levels in the serum and lipid peroxidation in the brain, as well as reducing the Nrf2 levels in the hippocampus. Moreover, pyridoxine deficiency reduces the proliferating cells and neuroblasts, probably by reducing BDNF levels. This result suggests that pyridoxine is an essential element in fighting neuronal death and in increasing the regenerative potentials after ischemia.

## 4. Materials and Methods

### 4.1. Experimental Animals

Male Mongolian gerbils (5 weeks of age) were purchased from Japan SLC, Inc. (Shizuoka, Japan). The animals were housed and cared based on the Guide for the Care and Use of Laboratory Animals (8th edition, 2011). Experimental protocols were approved by the Institutional Animal Care and Use Committee (IACUC) of Seoul National University (SNU-190408-2) on 14 May 2019.

After a week of acclimation, gerbils were fed with pyridoxine-deficient diet (PDD, D10001, Research Diets) and its control diet (CD, D15501R, control, Research Diets, New Brunswick, NJ, USA) for 56 days as described in the previous study [20] since the half-life of the elimination of pyridoxine exceeds 15 to 20 days [56]. All animals used in this study were sacrificed on the 56th day of diet feeding.

### 4.2. Ischemic Surgery

Animals were anesthetized with 2.5% isoflurane (Baxter, Deerfield, IL, USA) and then carotid arteries were isolated from adjacent tissue and occluded using aneurysm clips for 5 min as previously reported [57]. Blood flow through carotid arteries was monitored in the central artery of the retinae using an ophthalmoscope (HEINE K180^®^; HEINE Optotechnik, Herrsching, Germany). Body temperature (37 ± 0.5 °C) was regulated by a thermometric blanket under the monitoring using a rectal temperature probe (TR-100; Fine Science Tools, Foster City, CA, USA) until recovered from anesthesia. Two animals with incomplete ischemic induction were excluded. The sham group received the same procedures except for carotid artery occlusion.

### 4.3. Immunohistochemistry

To show the morphological evidence of the changes in neurons, astrocytes, and microglia in the hippocampus, immunohistochemical staining was conducted for NeuN, GFAP, and Iba-1, respectively, as previously described [20,57]. In addition, proliferating cells and neuroblasts were visualized with the immunohistochemistry of Ki67 and DCX. Briefly, animals (*n* = 5 per group) were anesthetized with a mixture of 75 mg/kg alfaxalone and 10 mg/kg xylazine on the 56th day of diet feeding, and blood was obtained by cardiac puncture in the right ventricle. Thereafter, animals were perfused transcardially, and the brain was coronally sectioned with a 30 μm thickness between 2.0 and 2.7 mm caudal to the bregma based on gerbil stereotaxic coordinates [58]. Four sections located 150 μm apart were selected and incubated with each antibody; mouse anti-NeuN antibody (1:1000; Merck Millipore, Temecula, CA, USA), rabbit anti-GFAP antibody (1:1000; Merck Millipore), rabbit anti-Iba-1 (1:500; Wako, Osaka, Japan), rabbit anti-Ki67 (1:1000; Abcam, Cambridge, UK), or rabbit anti-DCX (1:2000; Abcam). Immunoreaction was visualized with 3,3′-diaminobenzidine tetrachloride (Sigma, St. Louis, MO, USA) in 0.1 M Tris-HCl buffer (pH 7.2). Sections were dehydrated and mounted on gelatin-coated slides in Canada balsam (Kanto Chemical, Tokyo, Japan).

### 4.4. High-Performance Liquid Chromatography (HPLC) Analysis

Blood samples were obtained by cardiac puncture in gerbils for immunohistochemical staining described in Section 2.3. PLP and homocysteine levels were measured in the serum as described before [20]. Briefly, serum was injected onto a C_18_ reverse-phase column (250 mm × 4.6 mm, 5 μm; Agilent Technologies, Santa Clara, CA, USA) in an HPLC system (Agilent 1100 series, Agilent Technologies, Santa Clara, CA, USA) equipped with an electrochemical detector.

### 4.5. Malondialdehyde Assay

To elucidate the effects of pyridoxine deficiency on lipid peroxidation induced by ischemia, transient forebrain ischemia was induced with occlusion of common carotid arteries, and the animals (*n* = 5 per group) were sacrificed at 3, 12, and 24 h after ischemia/reperfusion on the 56th day of diet feeding. Both hippocampi were quickly removed, and MDA levels were measured with a Bioxytech MDA-586 kit (Oxis Research, Portland, OR, USA) as described in the previous study [59]. MDA levels were normalized to the protein concentration and the assay was triplicated.

### 4.6. Western Blot Analysis

Nrf2 and BDNF levels were assessed by Western blotting as described in the previous study [16]. Briefly, animals (*n* = 5 per group) were sacrificed 24 h after ischemia/reperfusion under deep anesthesia with a mixture of 75 mg/kg alfaxalone and 10 mg/kg xylazine on the 56th day of diet feeding. The left hippocampus was homogenized briefly, and the nuclear and cytosolic fractions were separated using extraction kits following the manufacturer’s instructions (Abcam). Homogenized proteins were loaded onto sodium dodecyl sulfate polyacrylamide gel electrophoresis. Thereafter, the gel was transferred onto a nitrocellulose membrane (Pall Crop, East Hills, NY, USA), and the membrane was incubated with rabbit anti-Nrf2 (1:1000, Abcam) and rabbit anti-BDNF (1:5000, Abcam). Protein bands were visualized using a chemiluminescence solution (GE Healthcare, Buckinghamshire, UK) and were normalized versus laminin A + C and β-actin levels, respectively, as demonstrated in the previous study [20].

### 4.7. Data Analysis

Four sections per antibody between 2.0 mm and 2.7 mm caudal to the bregma [27] were examined using an image analysis system and ImageJ software v. 1.5 (National Institutes of Health, Bethesda, MD, USA). Digital images of the whole dentate gyrus and midpoint of the CA1 region were captured with a BX51 light microscope (Olympus, Tokyo, Japan) equipped with a digital camera (DP72, Olympus). The number of NeuN- and Ki67-positive nuclei were counted in the hippocampal CA1 region and dentate gyrus, respectively, using an image analysis system (Optimas 6.5, CyberMetrics, Scottsdale, AZ, USA). Intensities for GFAP, Iba-1, and DCX were evaluated by ROD obtained after transforming the mean gray level using this formula: ROD = log(256/mean gray level). The ROD of background staining was determined in unlabeled portions of the sections using Photoshop CC software (Adobe Systems Inc., San Jose, CA, USA), and this value was subtracted to correct for nonspecific staining using ImageJ v. 1.50 software (National Institutes of Health, Bethesda, MD, USA). The data are expressed as the percentage of the vehicle-treated group values (set to 100%).

### 4.8. Statistical Analysis

The data obtained represent the mean with standard deviation. Differences among means were statistically analyzed using the two-way ANOVA test, followed by Bonferroni post-hoc tests. Statistical significance was considered at *p* < 0.05.
